# 1327. Human Rhinovirus Infection in Multiple Myeloma Patients: Effect on Morbidity and Mortality

**DOI:** 10.1093/ofid/ofab466.1519

**Published:** 2021-12-04

**Authors:** Lana Hasan, Juan Carlos Rico Crescencio, Mitchell Jenkins, Mary J Burgess

**Affiliations:** 1 University of Arkansas for medical Sciences, Little rock, Arkansas; 2 University of Arkansas for Medical Sciences, Little Rock, Arkansas; 3 Conway Regional Hospital, Conway, Arkansas

## Abstract

**Background:**

Human Rhinovirus (hRV) causes mild, primarily upper respiratory tract symptoms in immunocompetent hosts. However, in immunocompromised patients, it often progresses to a lower respiratory tract infection. Multiple myeloma (MM) patients are immunocompromised due to inherent immunodeficiency and exposure to biologic and chemotherapeutic agents. The complications of hRV infection in MM patients are not well known. In this study, we aim to identify the morbidity and mortality associated with hRV in MM participants.

**Methods:**

This was a retrospective study, using Arkansas Clinical Registry Database, which identified all MM patients diagnosed with hRV infection by nasopharyngeal multiplex polymerase chain reaction (PCR) in January-December 2019. Duplicates within 30 days were excluded. Patients were followed for 30 days after diagnosis. We assessed the need for hospitalization, intensive care unit (ICU) admission, oxygen administration, mechanical ventilation, and death. We collected their absolute neutrophil (ANC) and lymphocyte count (ALC) within three days of diagnosis and compared values using Mann-Whitney U test.

**Results:**

We identified 217 MM patients with hRV. Ninety (41%) had prior autologous stem cell transplant, 148 (68%) had received chemotherapy within 30 days. Ninety (41%) had chest imaging, with 11 (12%) having infiltrates. Out of the 217, 69 (31.9%) were admitted, with a mean length of stay of 3 days. 13% of the admitted patients were transferred to the ICU. 65.5% of the admitted patients needed oxygen, and two required mechanical ventilation. The mean ANC and ALC for the admitted group was 3.88 cells/µL and 1.22 cells/µL respectively, compared to 3.57 cells/µL and 1.07 cells/µL in the outpatient group, p=0.6 and 1. Five participants died.

**Conclusion:**

Human Rhinovirus infection in MM patients was associated with significant morbidity, including hospitalization, ICU care, supplemental oxygen requirement, and even mechanical ventilation in 2 patients. Death was observed within 30 days, although rarely. The mean ALC and ANC were not predictive of the severity of the disease. Recognizing hRV effects on morbidity and mortality could lead to earlier recognition and management of complications in MM patients.

**Disclosures:**

**All Authors**: No reported disclosures

